# A new genus and species of the family Pennellidae (Copepoda, Siphonostomatoida) infecting the Pacific viperfish *Chauliodus macouni*

**DOI:** 10.1051/parasite/2018003

**Published:** 2018-02-09

**Authors:** Susumu Ohtsuka, Dhugal J. Lindsay, Kunihiko Izawa

**Affiliations:** 1 Takehara Station, Setouchi Field Science Center, Graduate School of Biosphere Science, Hiroshima University, 5-8-1 Minato-machi, Takehara, Hiroshima 725-0024 Japan; 2 Research and Development Center for Submarine Resources, Environmental Impact Assessment Research Group, Japan Agency for Marine-Earth Science and Technology, 2-15 Natsushima-cho. Yokosuka, Kanagawa 237-0061 Japan; 3 Izawa Marine Biological Laboratory, 795-16 Kannonji, Tsu, Mie 514-0062 Japan

**Keywords:** Copepoda; deep-sea; Pennellidae; taxonomy

## Abstract

A new genus and species of pennellid copepod, *Protosarcotretes nishikawai* n. g., n. sp., is described on the basis of an ovigerous female infecting a Pacific viperfish *Chauliodus macouni* collected from the deep-waters of Suruga Bay, Japan. The new genus exhibits the most plesiomorphic states in the first to fourth legs of pennellids, and is differentiated from two closely related pennellid genera *Sarcotretes* and *Lernaeenicus* by the morphology of the oral appendages. Two species of the genus *Lernaeenicus* are transferred to the new genus as *Protosarcotretes multilobatus* (Lewis, 1959) n. comb. and *Protosarcotretes gnavus* (Leigh-Sharpe, 1934) n. comb. The host specificity and life cycle of deep-sea pennellids are discussed. *Sarcotretes scopeli* Jungersen, 1911 and *Cardiodectes bellottii* (Richiardi, 1882) show low differentiated host-specificity, while *P. nishikawai* seems to be limited to the Stomiidae, which are rare hosts of pennellids, in contrast to the Myctophidae family. In the Pennellidae family, two patterns of the life cycle are found: with or without naupliar stages.

## Abstract (fr)

Un nouveau genre et espèce de copépode Pennellidae, *Protosarcotretes nishikawai* n. g., n. sp., est décrit sur la base d’une femelle ovigère infectant le poisson-vipère du Pacifique *Chauliodus macouni* prélevé dans les eaux profondes de la baie de Suruga, au Japon. Le nouveau genre présente les états les plus plésiomorphes des appendices 1 à 4 des Pennellidae et se différencie de deux genres apparentés, *Sarcotretes* et *Lernaeenicus*, par la morphologie des appendices oraux. Deux espèces du genre *Lernaeenicus* sont transférées dans le nouveau genre comme *Protosarcotretes multilobatus* (Lewis, 1959) n. comb. et *Protosarcotretes gnavus* (Leigh-Sharpe, 1934) n. comb. La spécificité de l’hôte et le cycle de vie des Pennellidae profonds sont discutés. *Sarcotretes scopeli* Jungersen, 1911 et *Cardiodectes bellottii* (Richiardi, 1882) montrent une spécificité d’hôte peu différenciée, tandis que *P. nishikawai* semble être limité aux Stomiidae, rarement utilisés comme hôtes par les pennellidés, contrairement à la famille Myctophidae. Dans la famille Pennellidae, on retrouve deux types de cycle de vie, avec ou sans étapes nauplius.

## Introduction

Pennellid copepods are highly modified, meso- or ecto-parasitic copepods infecting marine fish and mammals as definitive hosts [8]. The life cycle of the family is complex, with some genera needing two hosts, while others require only a single host [7,8,20,24]. The intermediate hosts of *Cardiodectes* Wilson, 1917 and *Pennella* Oken, 1815, with two hosts each, are free-swimming molluscs [8,16,31,36]. Some species of the genera *Pennella*, *Peniculus* Nordmann, 1832 and *Lernaeenicus* Le Sueur, 1824 heavily parasitize wild and cultured commercially important fish and squids throughout the world’s oceans, presumably causing economic losses [3,20,28,31,32,33,34,35,38,48]. The genera *Cardiodectes* and *Sarcotretes* Jungersen, 1911 have been found on mesopelagic and bathypelagic fish [5,8,13,17,18,22,46,49].

**Figure 1:**
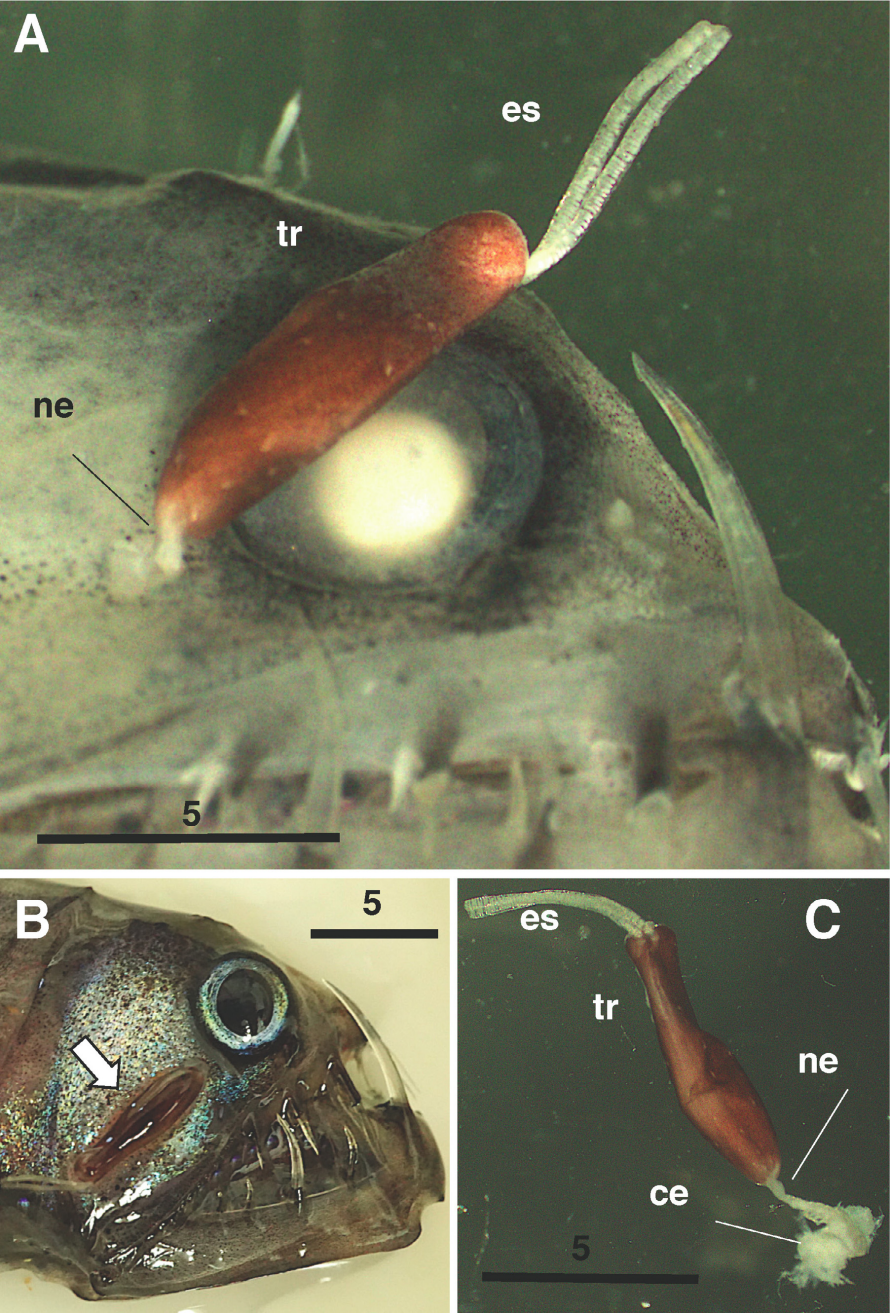
*Protosarcotretes nishikawai* n. g., n. sp., adult female (holotype). A. whole specimen, in-situ on host, after fixation; B. whole specimen (arrowed), in-situ on host, before fixation; C. whole specimen, dissected out of host. Abbreviations: ce: cephalothorax, es: egg string, ne: neck, tr: trunk. Scales in mm.

During a survey on the deep-water plankton of Suruga Bay, Japan, an undescribed pennellid copepod was discovered on the Pacific viperfish *Chauliodus macouni*, 1890, Bean (Fig. 1A, B). This animal generally resembles three pennellid genera, *Sarcotretes*, *Lernaeenicus* and *Peniculus*, placing it within the family Pennellidae as defined by Boxshall [4], although the first two genera appear taxonomically confused. In *Sarcotretes* and *Lernaeenicus*, the neck (see “ne” in Fig. 1) is composed of the first to fourth pedigerous somites, while in *Peniculus*, the fourth pedigerous somite is incorporated into the trunk [8]. According to the keys to pennellid genera provided by Kabata [23] and Boxshall & Halsey [8], a feature distinguishing these two genera is the presence (in *Sarcotretes*) or absence (in *Lernaeenicus*) of a middle constriction of the neck. However, this is not applicable to all species of the former. For example, *Sarcotretes longirostris* Ho, Nagasawa, & Kim, 2007 bears a slender neck without a constriction midway (see Fig. 1A in Ho et al. [17]). On the other hand, *Lernaeenicus* also seems to be a catch-all group when the morphological variability in the cephalosomes, abdomens and legs is considered. Some species of *Lernaeenicus* bear a well-developed abdomen, while in others it is highly reduced like in *Sarcotretes*. In many species, legs 3 and 4 are uniramous, while in *L. multilobatus* Lewis, 1959 they are biramous. Castro Romero [11] provided a different key to pennellid genera, and suggested that the key characteristics differentiating these two genera are the morphology of the cephalic holdfasts, proboscis and labium.

The present paper deals with the taxonomy of the undescribed pennellid copepod parasitizing the Pacific viperfish, and discusses the validity of the genera *Sarcotretes* and *Lernaeenicus*.

## Materials and methods

The present specimens (a parasitic copepod attached posterior to the right eye of its host fish) were captured in Suruga Bay (35°02.3’N, 138°40.5’E) between 12:21-13:51 on September 8, 2017 in an oblique tow (0-810 m depth) of an ORI net (335 μm mesh, 1.6 m mouth diameter) during cruise SRM17-9-VPR of the T/V Hokuto (Tokai University). The specimens were photographed live before being preserved in 99.5% ethanol (see Fig. 1). The host fish was identified as *Chauliodus macouni* Bean, 1890 by reference to Nakabo [29].

The parasitic copepod was removed from the host tissue and then partly dissected in lactophenol with a pair of fine needles under a dissecting microscope (SZX7, Olympus Co., Ltd.). The body and appendages were examined in lactophenol and drawn with the aid of a camera lucida attached to a compound microscope (BX53, Olympus Co., Ltd.). The specimens were deposited in the Kitakyushu Museum of Natural History and Human History (KMNH). Terminology follows Huys & Boxshall [19] and Ho et al. [17].

## Results

### Genus *Protosarcotretes* n. g.

urn:lsid:zoobank.org:act:C3402CB8-65B1-40A1-9A9A-EA03548D5E64

Order Siphonostomatoida Burmeister, 1835

Family Pennellidae Burmeister, 1835

*Type species*. *Protosarcotretes nishikawai* n. g., n. sp. (by monotypy).

*Other species*. *Protosarcotretes multilobatus* (Lewis, 1959) (new combination); *Protosarcotretes gnavus* (Leigh-Sharpe, 1934) (new combination).

*Etymology*. The new generic name is derived from *proto* (Greek prefixed, meaning primitive) and a closely related genus *Sarcotretes*, and refers to the primitive condition, especially in the segmentation and setation of legs 1–4, of the new genus. Gender masculine.

*Diagnosis*. Body straight, without brush-like structure on abdomen. Cephalothoracic holdfast represented by pair of lateral expansions. Oral cone weakly produced anteroventrally to form proboscis. Neck comprising pedigers 2–4, first urosomite and anterior part of trunk. Trunk cylindrical; abdomen highly reduced; caudal rami present, bilobate with 2 and 4 setae, respectively. Egg string uniseriate. Total length ca. 10 mm.

Antennule indistinctly 4-segmented. Antenna 3-segmented, heavily sclerotized; second segment produced at subterminal corner into stout triangular process; third segment curved inward to form subchela with process of preceding segment, bearing minute basal seta. Mandible simple stylet-like, with no teeth distally. Maxillule unilobate, inner lobe with 2 terminal setae; outer lobe absent. Maxilla 2-segmented; first segment with no accessory process; second segment bearing 4 rows of spinular prominences on calamus.

Legs 1–4 biramous; rami 2-segmented; armature elements shown in Table 1.

**Table 1:** Segmentation and setation of legs 1 to 4 of *Sarcotretes*, *Lernaeenicus* and *Protosarcotretes* n. g. Bold letters indicate differences among genera. Number in parentheses shows variation.

Genus	Leg	Protopod	Exopod	Endopod
*Sarcotretes*	1	**1-0**	I-1, I,I,5	0-1, **7**
	2	1-0	**I-1, I,I,5**	0-1, 7
	3	1-0	**0-0, I,I,4**	**absent**
	4	**absent**	**absent**	**absent**
*Lernaeenicus*	1	**1-1**	I-1, I,I,5	0-1, **7**
	2	1-0	**0(I)-1, I,6**	0-1, 7
	3	1-0	**0-0, I,5**	**absent**
	4	**1-0**	**0-0, I,4**	**absent**
*Protosarcotretes* n. g.	1	**1-1**	I-1, I,I,5	0-1, **8**
	2	1-0	**I-1, II,I,5**	0-1, 7
	3	1-0	**I-1, I,I,5**	**0-1, 4**
	4	**1-0**	**I-1, I,I,5**	**0-1, 3**

*Remarks*. Once both *Sarcotretes* and *Lernaeenicus* are rigidly defined, it is evident that the establishment of a new genus for the present material is warranted. However, since many taxa belonging to these genera were poorly described in the 18th and at the beginning of the 19th centuries, the definitions below are still tentative and await a complete revision (see Raja et al. [38]).

Adult females of *Sarcotretes* are relatively rigidly defined by the following synapomorphies in comparison with other closely related pennellid genera: (1) paired cephalic holdfasts expanded laterally, (2) oral cone moderately or highly developed, produced anteroventrally to form proboscis, (3) abdomen highly reduced, (4) caudal rami absent, (5) leg 3 uniramous, and leg 4 represented by vestige, (6) armature elements of legs 1–3 as presented in Table 1 (based on Uyeno et al. [46]), and (7) rudimentary outer spines present on first exopodal segments of legs 1 and 2. Based on observations of the labium by Castro Romero & Kuroki [12] and Castro Romero [11], those of *Sarcotretes* bear a pair of pad-like structures. The body length of adult females ranged from 13–85 mm [13,17,18,22,46,49]. This genus has so far accommodated the following four valid species: *S. eristaliformis* (Brian, 1908); *S. scopeli* Jungersen, 1911; *S. longrostris* Ho, Nagasawa, & Kim, 2007 and *S. umitake* Uyeno, Wakabayashi & Nagasawa, 2014. *Sarcotretes* mainly parasitizes deep-sea planktonic and benthic fish.

Adult females of *Lernaeenicus* are characterized by: (1) cephalothorax usually bearing 3 or more dorsal, simple or branching processes/knobs (at least one median and 2 lateral), which are sometimes heavily sclerotized, (2) oral cone moderately or highly produced anteroventrally, (3) abdomen elongate, (4) caudal rami present or absent, (5) first segment of maxilla with one or more processes, (6) legs 3 and 4 both retained, uniramous, (7) armature elements shown in Table 1 (based on Shiino [42,43], Kabata [23], Sebastian & George [41], Schram [39], Oldewage [30], and Knoff & Boeger [25]), and (8) rudimentary outer spine present on first exopodal segment of leg 1. In addition, Castro Romero [11] considered that the presence of a row of spinules or scale-like plates on the labium is important to define the genus. The body length is highly variable, ranging from 12 to 126 mm, [23,30,38,40,49]. According to Boxshall & Walter [9], 32 valid species are assigned to *Lernaeenicus*. However, it seems that *Lernaeenicus quadrilobatus* Yamaguti & Utiumi, 1953 infecting the blue lantern-fish *Diaphus coeruleus* (Klunzinger) is intermediate between *Lernaeenicus* and *Sarcotretes* on the basis of the morphology of the cephalothorax, abdomen and legs. *Lernaeenicus gracilis* (Heller, 1865), infecting the shallow-water carangid *Lichina amia* (Linnaeus), is also enigmatic, being similar to the new genus in having the cephalothorax with a pair of simple lateral expansions and a short abdomen. However, Heller [15] mentioned that the four pairs of legs below the neck are completely the same as in *L. monillaris* (=*Lernaeenicus sprattae*) with legs 3 and 4 uniramous. A taxonomic conclusion is pending until this species is redescribed in detail.

Raja et al. [38] summarized the host-parasite relationships of 13 species of *Lernaeenicus* occurring in the Indian Ocean: their hosts are restricted to shallow water families such as Blenniidae, Carangidae, Engraulidae, Hemiramphidae, Mugilidae, Nemipteridae, Polynemidae, and Scombridae.

The new genus described here shows many plesiomorphies in the oral cone and legs (see Boxshall [4]), but some states in the mandible, maxillule and maxilla can be regarded as apomorphic. Although *Lernaeenicus multilobatus* Lewis, 1959 parasitic on the angler-fish *Gigantactis* sp. (Gigantacinidae), was poorly described by Lewis [27], it can be assigned to the new genus by: (1) the holdfast composed of a pair of cephalothoracic lateral expansions, (2) the abdomen being highly reduced, and (3) leg 4 being biramous. *Lernaeenicus gnavus* Leigh-Sharpe, 1934 was poorly described on the basis of a single adult female with a damaged cephalothorax, in which the oral cone cannot be seen in Fig. 35 of the original description [26]. However, the morphological and ecological features suggest that it is probably assignable to the new genus we describe: (1) the abdomen is reduced; (2) the body length is about 10 mm, regardless of the damaged cephalothorax; (3) the host fish *Polyipnus spinosus* Günther belongs to the deep-sea family Sternoptychidae.

An evolutionary trend in reduction of segmentation of the legs is distinct in adult females of the *Protosarcotretes*-*Sarcotretes*-*Lernaeenicus* lineage (present study). Similar patterns can be found in the legs of parasitic copepod families such as Chondracanthidae, Pandaridae, and Hatschekiidae [23]. Generally, anterior legs are relatively conserved and show full segmentation in their rami, while posterior legs tend to have the number of segments reduced, finally leading to a vestigial condition (Table 1).

### *Protosarcotretes nishikawai* n. g., n. sp.

urn:lsid:zoobank.org:act:BA8F41A1-F58B-4922-81AA-3EA509AD9124

(Figs 1, 2)

**Figure 2:**
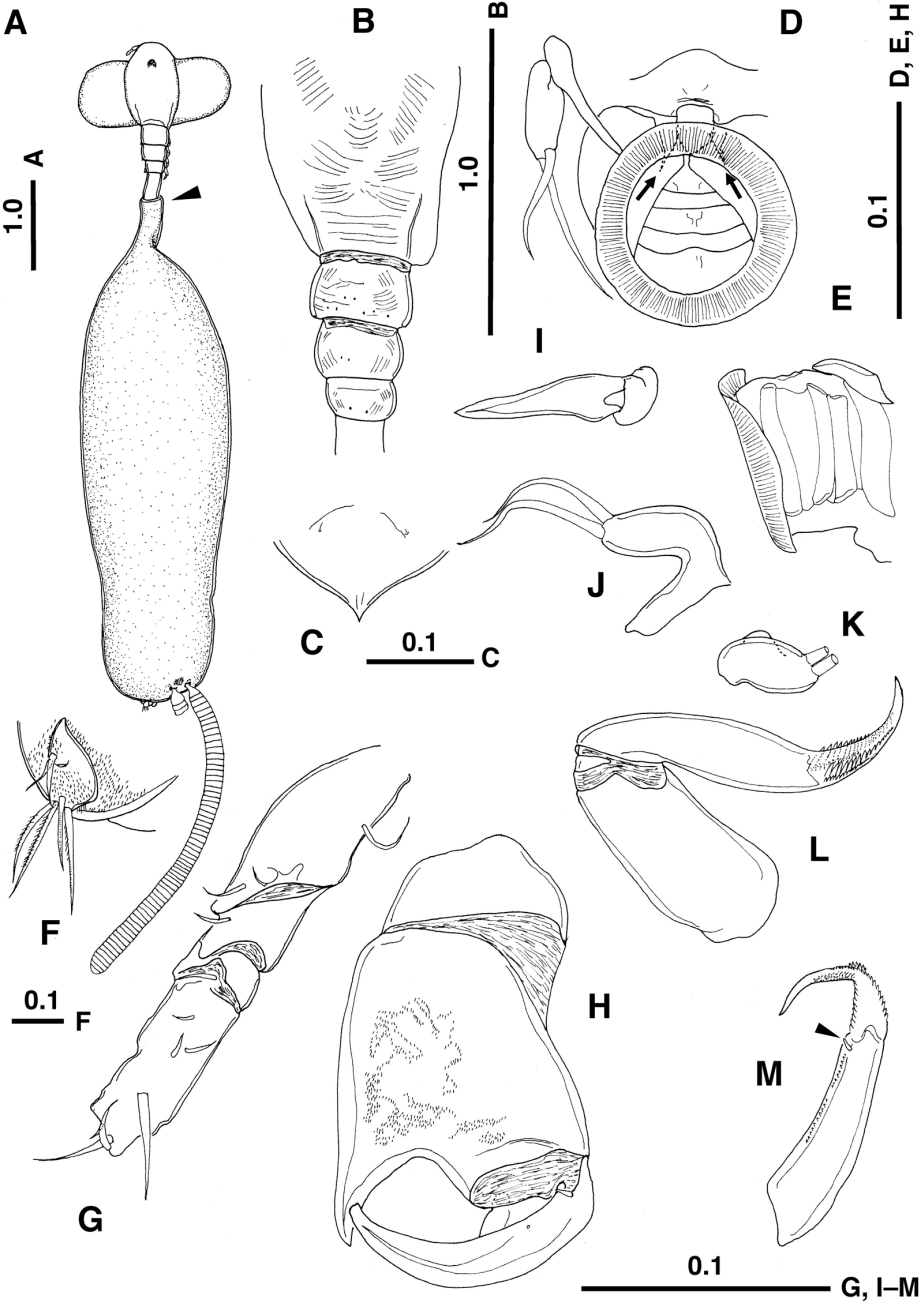
*Protosarcotretes nishikawai* n. g., n. sp., adult female (holotype). A. Habitus, dorsal view (trunk twisted), embedded part anterior to arrowhead; B. Pedigers 1–4, dorsal view; C. Rostrum; D. Oral cone, ventral view, pair of buccal stylets arrowed; E. Oral cone, lateral view; F. Caudal ramus; G. Antennule; H. Antenna; I. Mandible; J. Maxillule; K. Maxillule, setae omitted; L. Maxilla; M. Terminal segment of maxilla, canna arrowed. Scales in mm.

*Type-material*. Holotype, one ovigerous female infecting the Pacific viperfish *Chauliodus macouni* (standard length 127 mm) (KMNH VR 110,001) collected from depths of 0–810 m, Suruga Bay (35°02.3’N, 138°40.5’E) between local time 12:21–13:51 on September 8, 2017, cephalothorax partly dissected and mounted on a glass slide, body in vial, KMNH IvR 500,945.

*Type-locality.* Suruga Bay (35°02.3’N, 138°40.5’E), off Japan.

*Host and attachment site*. *Chauliodus macouni* Bean; attachment site: skin posterior to right eye.

*Etymology*. The new specific name is in honor of Professor Jun Nishikawa (Tokai University) who was helpful in collection of the present parasitic copepod during his research cruise in September 2017.

*Description*. Adult ovigerous female. Body (Figs 1, 2A) consisting of expanded cephalosome, relatively short neck and cylindrical trunk. Trunk tinged dark brown before fixation (Fig. 1B), and bearing white spots sparsely after fixation (Fig. 1A, C). Total length 10.6 mm from anterior tip of cephalosome to posterior end of caudal ramus, excluding setae. Parts anterior to genital complex (trunk) embedded in host tissue (arrow in Fig. 2A). Integument of dorsal side of posterior parts of cephalothorax and pedigers 2–4 finely wrinkled (Fig. 2B). Cephalothorax expanded laterally, forming paired holdfasts covered with thin cuticular membrane. Rostrum (Fig. 2C) pointed at tip, with pair of fine hair-like sensilla anteriorly. First pediger incompletely incorporated into cephalon. Naupliar eyes present (Fig. 2A). Oral cone (Fig. 2D, E) produced ventrally, not forming elongate proboscis, with four ring-like structures (Fig. 2D); pair of buccal stylets positioned anteriorly (arrowed in Fig. 2D). Neck comprising pedigers 2–4, first urosomite and anterior part of trunk, 1.4 mm in length. Trunk (Figs 1C, 2A) 7.2 mm in length, about 2.2 times as long as cephalothorax and neck combined; paired gonopores located subterminally; abdomen highly reduced, furnished with minute prominences; caudal ramus (Fig. 2F) bilobate, outer and inner lobes bearing 2 and 4 setae, respectively. Egg string (Figs 1, 2A) straight, uniseriate, containing 65 eggs in left sac.

Antennule (Fig. 2G) incompletely 4-segmented, possibly many setal elements missing, probably during dissection. Antenna (Fig. 2H) heavily chitinized, 3-segmented; second segment ornamented with minute prominences on surface, remarkably produced into triangular subterminal process on inner margin, opposing tip of subchela formed by third segment; third segment curved inward, with minute basal element on anterior surface. Mandible (Fig. 2I) simple stylet with no teeth terminally. Maxillule (Fig. 2J, K) unilobate, inner lobe with two terminal setae of unequal length; outer lobe absent. Maxilla (Fig. 2L, M) 2-segmented; first segment (lacertus) unarmed; second segment (brachium) reflexed, with terminal third (calamus) smoothly curved inward, tapering distally, having 2 rows of spinular prominences on each side; canna subterminally located on second segment, small (arrow in Fig. 2M).

**Figure 3:**
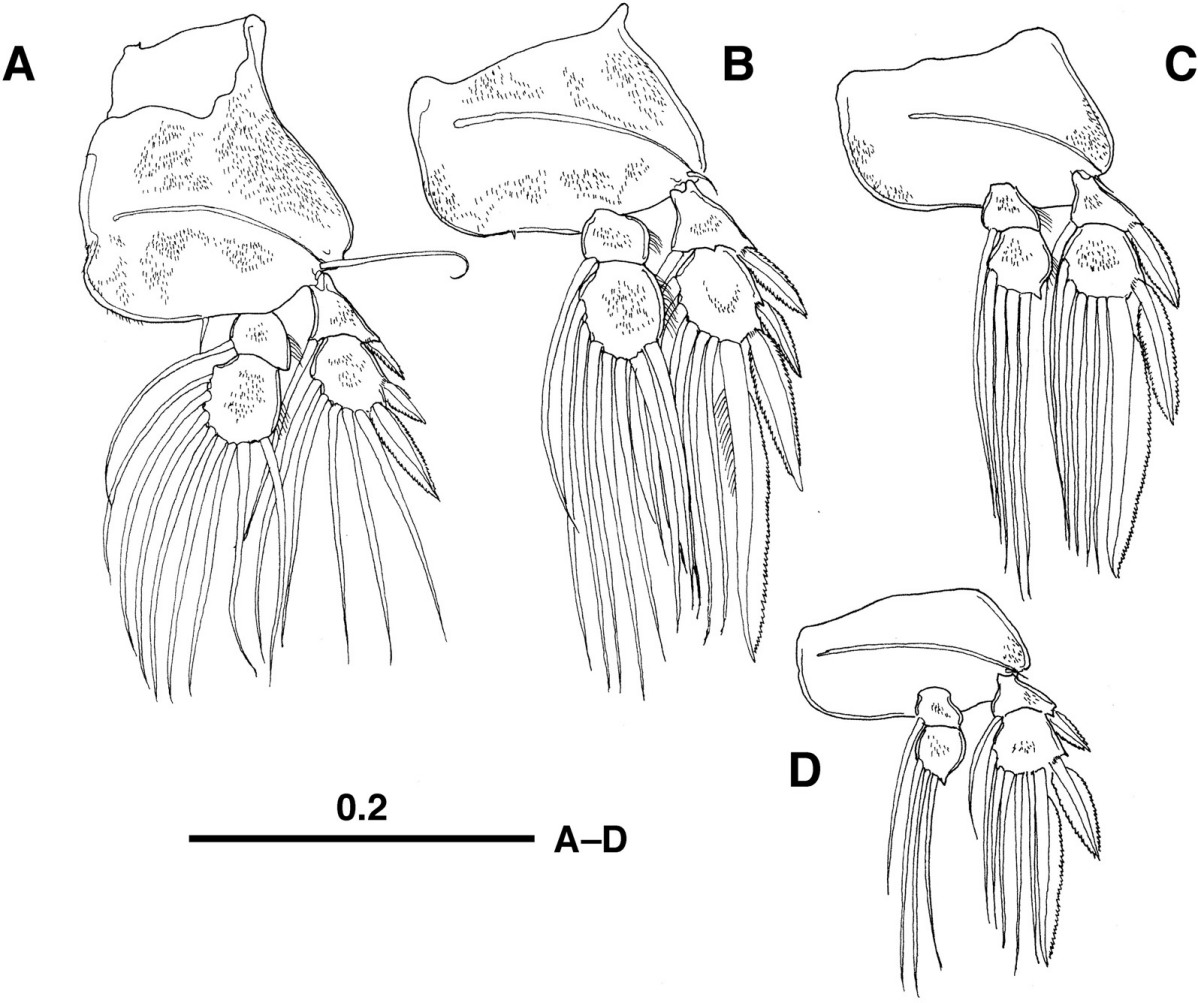
*Protosarcotretes nishikawai* n. g., n. sp., adult female (holotype). A. Leg 1, anterior surface; B. Leg 2, anterior surface; C. Leg 3, anterior surface, D. Leg 4, anterior surface. Scales in mm.

Legs 1-4 (Figs 3A–D) biramous, with 2-segmented rami; armature elements shown in Table 1; protopods with suture between coxa and basis distinctly visible; protopod and rami sparsely ornamented with minute spinules on surface.

*Remarks*. The new species is easily distinguished from its poorly described congener, *P. multilobatus* (Lewis, 1959) by the morphology of the holdfast: simple in the former and ramified in the latter. It differs from *P. gnavus* (Leigh-Sharpe, 1934) by the relative length of the trunk to the cephalothorax and neck combined (2.2 times in *P. nishikawai* n. sp. vs ca. 0.7 in *P. gnavus*).

## Discussion

Members of the parasitic family Pennellidae have successfully colonized the deep-sea [5,8,49]. Colonization of pennellids into the deep-sea seems to have occurred repeatedly, because the most basal genus *Peniculus* is a shallow-water taxon [4,23,47] and more derived groups are composed of a mixture of shallow- and deep-water taxa [4,23]. Host-parasite relationships in deep-sea taxa in the family are shown in Table 2. *Sarcotretes* and *Protosarcotretes* seem to be limited to deep waters, while only a few members of *Lernaeenicus* and *Cardiodectes* infect deep-sea fish. As already pointed out by Boxshall [5] and Boxshall & Halsey [8], *Sarcotretes scopeli* and *Cardiodectes bellottii* (Richiardi, 1882)  (as *C. medusaeus* (Wilson, 1908) exhibit low host-specificity, utilizing a wide range of fish families or genera. *Sarcotretes scopeli* infects eight families of fish, while *C.* *bellottii* parasitizes only Myctophidae. The Stomiidae host family utilized by *P. nishikawai* has only rarely been reported as a host of pennellids in contrast to the  family Myctophidae. It is interesting to note that Stomiiformes is generally thought to be basal relative to the Myctophidae [45], mirroring the condition in their copepod parasites. Visual observations with a Remotely-Operated Vehicle (ROV) have clearly recorded ectoparasitism of siphonostomatoid copepods such as Lernaeopodidae and Sphyriidae on deep-sea demersal fish, but not yet for Pennellidae [37]. This may partly be due to the relatively small-size of their bodies and partly due to their low abundances in the deep-sea.

**Table 2:** Host-parasite relationships of pennellid copepods infecting deep-sea fish. Scientific names of fish hosts are based on FishBase (2017) [14].

Parasitic copepod	Host family	Host species	Reference
*Sarcotretes scopeli* Jungersen, 1911	Myctophidae	*Benthosema glaciale* (Reinhardt)*, Diogenichthys atlanticus* (Tåning), *Electrona carlsbergi* (Tåning), *Gonichthys cocco* (Cocco), *Gymnoscopelus nicholsi* (Gilbert), *Gymnoscopelus* *piabilis* (Whitley), *Protomyctophum choriodon* Hulley, *Protomyctophum tenisoni* (Norman), *Krefftichthys anderssoni* (Lönnberg), *Lampichthys procerus* (Brauer), *Metelectrona ventralis* (Becker), *Myctophum punctatum* Rafinesque, *Notoscopelus resplendens* (Richardson), *Protomyctophum bolini* (Fraser-Brunner), *Protomyctophum choriodon* Hulley, *Protomyctophum tenisoni* (Norman), *Symbolophorus evermanni* (Gilbert)	[9,13,18,22,49]
	Melamphaidae	*Scopeloberyx malayanus* (Weber), *Scopeloberyx opisthopterus* (Parr), *Scopeloberyx robustus* (Günther)	
	Sternoptychidae	*Polyipnus asteroides* Schultz, *Sternoptyx diaphana* Hermann	
	Gempylidae	*Gempylus serpens* Cuvier	
	Gonostomatidae	*Cyclothone atraria* Gilbert	
	Macrouridae	*Hymenogadus gracilis* (Gilbert & Hubbs)	
	Melanocetidae	*Melanocetus johnsonii* Günther	
	Stomiidae	*Photostomias tantillux* Kenaley	
			
*Sarcotretes eristaliformis* (Brian, 1908)	Sternoptychidae	*Sternoptyx diaphana* (Hermann), *Sternoptyx obscura* Garman, *Sternoptyx pseudobscura* Baird	[9,13,18]
	Macrouridae	*Hymenocephalus striatissimus* Jordan & Gilbert, *Nezumia bairdii* (Goode & Bean)	
	Eurypharyngidae	*Eurypharynx pelecanoides* Vaillant	
	Gonostomatidae	*Sigmops gracilis* (Günther)	
	Ipnopidae	*Bathypterois dubius* Vaillant	
	Myctophidae	unidentified myctophids	
	Stomiidae	*Malacosteus niger* Ayres	
			
*Sarcotretes longirostris* Ho et al., 2004	Nomeidae	*Psenes pellucidus* Luüken	[17]
			
*Sarcotretes umitake* Uyeno et al., 2014	Macrouridae	*Coelorinchus jordani* Smith & Pope	[46]
			
*Lernaeenicus gonostonae* Kensley & Grindley, 1973	Gonostomatidae	*Sigmops elongatus* (Günther)	[9]
			
*Lernaeenicus quadrilobatu*s Yamaguti & Utiumi, 1959	Myctophidae	*Diaphus caeruleus* (Klunzinger)	[50]
			
*Protosarcotretes nishikawai* n. g., n. sp.	Stomiidae	*Chauliodus macouni* Bean	Present study
			
*Protosarcotretes gnavus* (Leigh-Sharpe, 1934)	Sternoptychidae	*Polyipnus spinosus* Günther	[26]
			
*Protosarcotretes multilobatus* (Lewis, 1959)	Gigantactinidae	*Gigantactis* sp.	[27]
			
*Exopenna crimmeni* (Boxshall, 1986)	Moriidae	*Antimora rostrata* (Günther)	[4]
			
*Cardiodectes bellottii* (Richiardi, 1882)	Myctophidae	*Benthosema glaciale, Ceratoscopelus townsendi* (Eigenmann & Eigenmann)*, Ceratoscopelus warmingii* (Lütken), *Diaphus theta* Eigenmann and Eigenmann, *Diaphus suborbitalis* Weber, *Gonichthys cocco* (Cocco), *Lampadena* cf. *dea*, *Lampanyolodes hectoris* (Günther), *Myctophum affine* (Lütken)*, Nannobrachium leucopsarum *(Eigenmann & Eigenmann), *Nannobrachium ritteri* (Gilbert)*, Parvilux ingens* Hubbs and Wishner, *Stenobrachius leucopsarus* (Eigenmann & Eigenmann), *Symbolophorus californiensis* (Eigenmann & Eigenmann)	[6,9,49]
			
			
*Cardiodectes cristatus* Shiino, 1958	Myctophidae	*Diaphanus suborbitalis* (as *D. glandulifer*)	[42]
			
*Cardiodectes krishnai* Sebastian, 1968	Phosichthyidae	*Vinciguerria luccetia* (Garman)	[9]
			
*Cardiodectes longicervicus* Shiino, 1958	Myctophidae	*Myctophum apserum* Richardson (as *Dasiscopelus asper* [sic])	[42]
			
*Ophiolernaea longiceps* Shiino, 1958	Sternoptychidae	*Polyipnus spinifer* Borodulina	[42]
			
*Parina myctophi* Kazachenko & Avdeev, 1977	Myctophidae	*Myctophum spinosum* (Steindachner)	[9]

The life cycle of *Protosarcotretes* is unknown, but can be deduced on the basis of that of other pennellids, especially *Lernaeenicus sprattae* (Sowerby, 1806) [1,7,10,16,20,24,36,39,40,41,44,49]. Basal pennellid groups such as *Peniculus*, *Sarcotretes*, *Lernaeenicus* [4,23] and *Protosarcotretes* may be characterized by the possession of a single host [7,20,23]. However, the number of developmental stages depends on the taxon. In *Peniculus,* *Lernaeenicus* and *Peroderma* two patterns with or without naupliar stages are recognized. i.e., 2 nauplii, 1 copepodid, 4 chalimi, and adult (in*L. sprattae*), and 1 copepodid, 4 chalimi, and adult (in *Peroderma cylindricum* Heller, 1868, *Peniculisa shiinoi* Izawa, 1965, and *Peniculus minuticaudae* Shiino, 1958) [2,20,21]. In *P. cylindricum* and *P. shiinoi*, only hatching stages were observed [2,21], while in *L. sprattae* and *P. minuticaudae* all post-embryonic developmental stages were fully described [20,39]. In deep-sea pennellids, the hatching stage is an infective copepodid in *C. bellottii* (Richiardi, 1882) [36], but is unknown in *Sarcotretes* and *Protosarcotretes*. Clarification of the life cycle would be possible if embryos developed to before the hatching stage were to be found inside the egg strings.
